# Modulation of Macrophages M1/M2 Polarization Using Carbohydrate-Functionalized Polymeric Nanoparticles

**DOI:** 10.3390/polym13010088

**Published:** 2020-12-28

**Authors:** Raquel G. D. Andrade, Bruno Reis, Benjamin Costas, Sofia A. Costa Lima, Salette Reis

**Affiliations:** 1 LAQV, REQUIMTE, Departamento de Ciências Químicas, Faculdade de Farmácia, Universidade do Porto, Rua de Jorge Viterbo Ferreira, 228, 4050-313 Porto, Portugal; up201305657@fc.up.pt; 2 Centro Interdisciplinar de Investigação Marinha e Ambiental (CIIMAR), Universidade do Porto, Avenida General Norton de Matos, S/N, 4450-208 Matosinhos, Portugal; breis@ciimar.up.pt (B.R.); bcostas@ciimar.up.pt (B.C.); shreis@ff.up.pt (S.R.); 3 Instituto de Ciências Biomédicas Abel Salazar (ICBAS-UP), Universidade do Porto, Rua de Jorge Viterbo Ferreira n° 228, 4050-313 Porto, Portugal

**Keywords:** glyconanoparticles, immunotherapy, infectious diseases, mannose receptors, nutraceuticals

## Abstract

Exploiting surface endocytosis receptors using carbohydrate-conjugated nanocarriers brings outstanding approaches to an efficient delivery towards a specific target. Macrophages are cells of innate immunity found throughout the body. Plasticity of macrophages is evidenced by alterations in phenotypic polarization in response to stimuli, and is associated with changes in effector molecules, receptor expression, and cytokine profile. M1-polarized macrophages are involved in pro-inflammatory responses while M2 macrophages are capable of anti-inflammatory response and tissue repair. Modulation of macrophages’ activation state is an effective approach for several disease therapies, mediated by carbohydrate-coated nanocarriers. In this review, polymeric nanocarriers targeting macrophages are described in terms of production methods and conjugation strategies, highlighting the role of mannose receptor in the polarization of macrophages, and targeting approaches for infectious diseases, cancer immunotherapy, and prevention. Translation of this nanomedicine approach still requires further elucidation of the interaction mechanism between nanocarriers and macrophages towards clinical applications.

## 1. Introduction

Nanomedicine aims to improve health and life welfare with nanosized materials. Nanoparticles can be designed for drug delivery by modulating surface properties and composition to improve therapeutic effect and targeting specificity. Active targeting can be obtained with surface functionalization of the nanoparticles using specific ligands to reach the target of interest [1]. Taking advantage of this receptor-mediated specificity will reduce toxicity and side-effects to healthy tissues.

Macrophages are innate immune cells widely present in the body acting to maintain homeostasis and to resist pathogen invasion [2]. Macrophages are distributed according to their functions, surface-expressed markers, and secreted cytokines in M1/M2-polarized phenotypes. However, the simplicity of M1/M2 dichotomy of macrophage activation is too broad to explain all the actual states of the macrophages as a response to several stimuli. To have a proper description of the macrophage activation, it is generally accepted to include information on macrophage source, type of activators, and markers [3]. An imbalance in the M1/M2 ratio weakens the immune response and leads to inflammation. Hence, macrophages constitute an important player in the therapeutic strategies against infections, inflammatory conditions, and cancer. Receptors frequently expressed on the surface of macrophages constitute a potential target for nanomedicine-based approaches. Macrophage scavenger receptor, Toll-like receptors, glucan receptor, folate receptor, and mannose receptor are among the most used surface receptors of macrophages [4]. Mannose receptor (MR) is composed by several domains that allows recognition to various molecules of the carbohydrate family and contributes to receptor-mediated endocytosis. Upon internalization, nanocarriers can elicit macrophage polarization in vivo. Different types of polymeric based carriers (e.g., nanoparticles, micelles, dendrimers) are emerging as macrophage-targeted delivery systems [5]. Nanocarriers can also reset the macrophage activation state, as it is the case of the conversion of M2 phenotype to M1 in tumor-associated macrophages [6]. Understanding the interaction mechanisms between nanoparticles and macrophages is essential to a successful and effective nanocarrier’s design towards a therapeutic or prevention strategy.

## 2. Polymeric Nanoparticles as Biomedical Delivery Devices

Over the past few decades, the development of new strategies that surpasses the problems associated with conventional diagnosis and therapies have gained great importance on the scope of nanomedicine. One of the main goals in this field is to design nanoparticles capable of a targeted delivery and controlled release of bioactive compounds to a specific site, increasing its therapeutic effect while minimizing its side effects [7,8]. Several types of nanoparticles can be prepared from different building blocks like lipids, proteins, metals, and polymers [7,8,9]. Polymeric nanoparticles have gained great importance as biocompatible drug delivery systems given their simplicity and low-cost production [10]. The use of polymeric nanoparticles in drug delivery has many advantages over the use of other types of nanocarriers: a growing choice of biodegradable and biocompatible polymers, higher encapsulation efficiencies, higher stability in physiological conditions, improved drug bioavailability, and simpler preparation (for more detailed information on the synthesis methods see ref. [11]).

The design of drug delivery systems needs to consider several characteristics, namely, hydrophobicity, size, surface charge, biological interactions/toxicity, and biodegradability. A wide variety of natural or synthetic polymers are available for the preparation of the nanoparticles [12,13]. To produce nanoparticles, the most commonly used natural polymers include chitosan, a linear polysaccharide extracted from the exoskeletons of marine crustaceans [14], alginate that is isolated from brown algae [15], and gelatin obtained by hydrolyzed collagen [16]. Natural polymers have the advantage of combining biological properties like mimicking the extracellular matrix, allowing to sustain cell growth in tissue engineering applications, and tunable mechanical properties like stimuli-responsiveness, degradation, swelling, and crosslinking capabilities [13,14,15]. However, the application of natural polymers is often hampered by contaminants and batch-to-batch variability. Other constraints involve low hydrophobicity that compromises lipophilic drugs encapsulation, and a rapid drug release from the matrix [17,18].

Limitations of natural polymers can be overcome with the use of synthetic polymers, which are more reproducible in manufacture and more stable. Polymeric nanoparticles obtained with synthetic polymers allow drug-controlled release for a period of days up to several weeks [18]. Drawbacks associated with these type of nanoparticles involve their limited aqueous solubility and the need of surfactants to form stable suspensions [19]. The outcome of the nanoparticle as a drug delivery system can be modulated in the composition not only in the nature of the polymer, but also in the molecular weight, copolymer composition, and selected surfactant. To produce a targeted drug release within the body, the nanoparticles can be considered with additional properties to respond to external or internal stimuli such as redox state or pH [20]. Polylactic acid (PLA), poly(glycolic acid) (PGA), and their copolymers (PLGA) represent the most extensively used and studied synthetic polymers for drug delivery [21,22,23]. The presence of ester linkages in their backbones make these polyesters biodegradable. In fact, in a living organism, these polymers suffer a hydrolysis, and the resulting products are easily metabolized in the Krebs cycle and eliminated as carbon dioxide and water [17]. Also widely applied in the production of nanoparticles is poly-ɛ-caprolactone (PCL) that allows a slow degradation rate in comparison with PLA and PLGA, and thus is more adequate for long-term drug delivery. Poly(alkylcyanoacrylate) (PACA) is an interesting polymer whose properties can be controlled by the side of the introduced chains, being that the longer the side-chains the longer the half-life of the nanoparticles [17].

**Figure 1:**
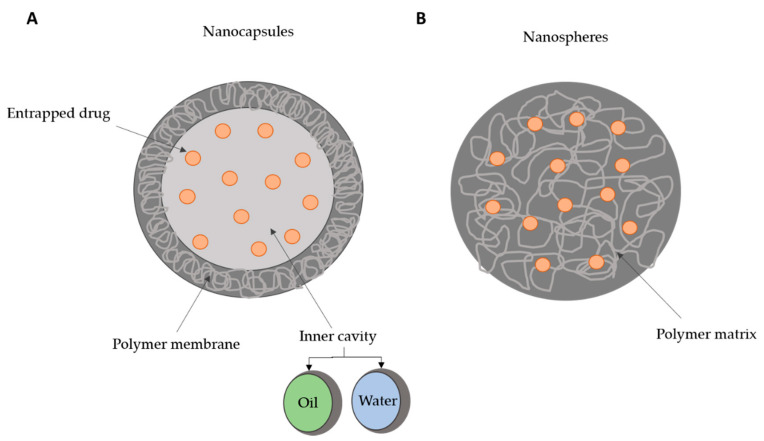
Schematic representation of the two types of polymeric nanoparticles: nanocapsules (**A**) and nanospheres (**B**). Nanocapsules comprise an inner cavity, composed of water or a semi solid (oil), and covered with a polymer membrane, while in nanospheres the entire mass is a polymer matrix. Drug molecules can be entrapped in both types of nanoparticles.

Depending on the preparation method, used polymers and desired application, different polymeric nanocarriers can be obtained such as polymer-drug conjugates, polymeric micelles, polymeric nanogels, and dendrimers [24]. Two types of polymer nanoparticles can be obtained for drug delivery: nanocapsules, composed of a liquid or semisolid core covered by a polymer membrane; or nanospheres that consist in a solid polymer matrix [11,12,23,24,25]. The drugs can be either entrapped in nanoparticles or adsorbed at the surface. In nanocapsules, the drug can be encapsulated in the inner core, while in nanospheres it is uniformly dispersed in the polymer matrix (Figure 1). These represent versatile tools for surface modification, as well as shape, size, and even optical characteristics. In nanomedicine, core–shell polymeric nanoparticles are also interesting, as the polymeric platform allows a second shell, usually a solid, which may confer smart properties to the nanoparticle (e.g., pH sensitive, thermo- and enzyme-responsive) [26,27,28].

## 3. Production Methods for Polymeric Nanoparticles and Surface Properties Modifications

**Figure 2:**
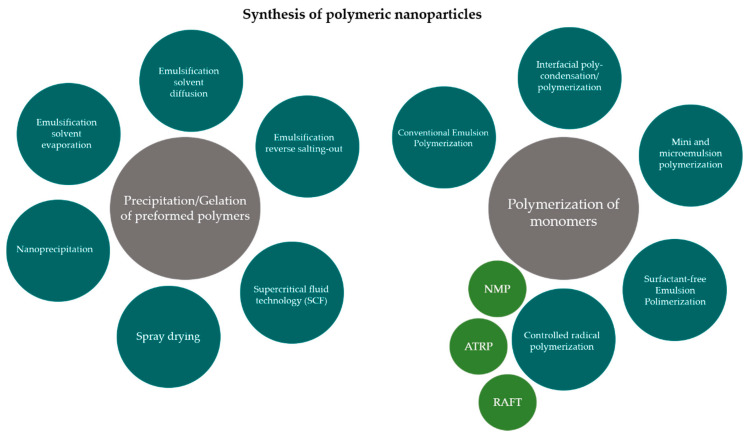
Diagram representing the options of production methods to obtain polymeric nanoparticles. Abbreviations: NMP (nitroxide-mediated polymerization); ATRP (atom transfer radical polymerization); RAFT (reversible addition and fragmentation transfer chain polymerization).

Currently, there are several methods developed and well-implemented for the preparation of polymeric nanoparticles. At first, one needs to ponder on (i) the physicochemical properties of the bioactive compound to be delivered, (ii) the nature and type of polymer, (iii) the target and biological environment, and (iv) the administration route. Based on this information it is possible to select the most adequate production method among emulsification-solvent evaporation, nanoprecipitation, emulsification reverse salting-out, and emulsification solvent diffusion. These polymerization processes allow production of nanoparticles with control of physicochemical and biological properties of the nanoparticles that are formed (Figure 2). At least two steps are involved in these conventional production methods: (i) polymer dissolution in an organic solvent followed by emulsification in an aqueous phase, and (ii) solvent evaporation to obtain the nanoparticles [13,29]. Polymeric nanoparticles can also be produced using monomers in an emulsion or as a micellar suspension by interfacial poly-condensation [13,17].

Hydrophilicity of the drug delivery systems represents an important feature to be considered for biological application. In fact, upon intravenous administration, hydrophobic nanoparticles are taken as foreign and the organism removes them from circulation to the excretion organs (liver, spleen, and lymph nodes) using the mononuclear phagocytic system [30]. If the intended treatment targets one of these organs, hydrophobic nanoparticles are the best solution. When aiming different targets, systemic circulation needs to occur, so the delivery systems reaches the diseased site. In this case, the surface of the nanoparticles must be modified with hydrophilic polymers to prevent the action of the mononuclear phagocytic system and phagocytosis. Hydrophilic nanoparticles will have long circulation times and reduced nonspecific distribution [31,32]. The list of hydrophilic polymers is long and include polyethylene glycol (PEG), poly-vinyl pyrrolidone (PVP), pluronics (poly-ethylene oxides), poloxamers, vitamin E TPGS, polysorbate 20, polysorbate 80, and polysaccharides (e.g., dextran) [33]. A protective layer can be obtained at the surface of the nanoparticles with these hydrophilic compounds, by adsorption or grafting shield groups. In some cases, PEG can be incorporated as copolymer [30,34,35]. The most used hydrophilic polymer for nanoparticles’ surface modification is PEG. The nature (flexible chains) and the physicochemical (hydrophilicity) features of this polymer as well as the presence of functional groups able to prevent plasma proteins binding are the reasons for this success [36]. A significant decrease in the opsonization and macrophage internalization of nanoparticles was observed with PEG coating, which lead to an enhanced long-term blood circulation. PEGylated nanoparticles promote a higher drug uptake by target tissues when compared to non-PEGylated ones [37,38,39].

In sum, a crucial feature of polymeric nanoparticles is their surface modification in order to improve drug delivery. On the one hand, the addition of a stealth layer (PEG, PVA, polysorbate) at the surface of nanoparticles allows an increased blood circulation time, avoiding the binding of opsonins and the rapid clearance from the mononuclear phagocytic system, and on the other hand, the functionalization at the surface with targeting ligands (proteins, peptides, antibodies [40,41,42]) improves the specificity of the treatment [43,44].

## 4. Carbohydrate-Functionalized Polymeric Nanoparticles 

As stated before, polymeric nanoparticles have excellent features that make them promising delivery systems for therapeutic applications. A higher specificity of drug delivery to a certain site of action is achieved when targeting ligands are incorporated in these nanocarriers. The functionalization of nanoparticles with carbohydrates, also known as glyconanoparticles, plays a key role in receptor-mediated delivery, as it allows to establish specific interactions with carbohydrate-binding proteins (lectins) [45,46]. Besides molecular recognition, sugars can act as colloidal stabilizers [47], reduce toxicity [48] and immunogenicity [49] and unlike PEG, increase circulation time in the bloodstream without compromising cellular uptake [50]. 

**Figure 3:**
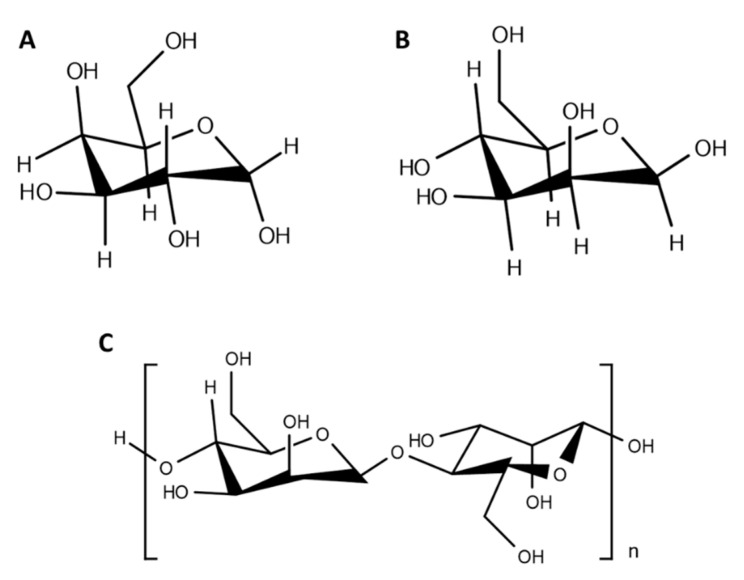
Chemical structure of some carbohydrates commonly used to produce glyconanoparticles. (**A**) Galactose; (**B**) mannose; (**C**) mannan.

Glycopolymers can be prepared either by post-polymerization modification, which consists in the functionalization of a preformed polymeric backbone, or in the polymerization of glycosylated monomers [51,52] that can be performed by several synthetic routes that provide controllable architectures, stereochemistry, and molecular weights, such as free radical polymerization (ring-opening polymerization (ROP)), ionic polymerization, controlled radical polymerization (nitroxide-mediated polymerization (NMP), atom transfer radical polymerization (ATRP), reversible addition fragmentation chain transfer (RAFT), and enzyme-mediated polymerization [51,53,54,55]. Here, we will focus on the post-functionalization of polymeric nanoparticles with carbohydrates, as it allows the attachment of pendant carbohydrate moieties (Figure 3), making it ideal for targeted delivery.

The coupling of a ligand to a nanoparticle can be achieved either by electrostatic interactions or by covalent conjugation strategies [56,57]. The last requires the presence of reactive functional groups (amine, carboxyl, sulfhydryl, hydroxyl, azide-reactive groups) at the surface of nanoparticle that enable conjugation with ligands [58]. A very popular method used for chemical conjugation is the carbodiimide method, which consists of the activation of carboxylate functional groups that react with primary amines to form amide bonds [58,59]. In this case, a direct conjugation is performed, but sometimes linkers are used. For instance, Kim and collaborators used *N,N*′-dicyclohexyl carbodiimide (DCC) for a two-step coupling reaction of a galactose moiety to polymeric nanoparticles composed of cholic acid and diamine-terminated poly(ethylene glycol) as a linker [60]. Palmioli and co-workers also described the functionalization of PLGA with sugar entities bearing a 2-(2-aminoethoxy)ethanol linker through amide bond using *N,N*′-diisopropylcarbodiimide and NHS [61]. 

Crucho and colleagues produced a polymeric conjugate composed of PLGA modified with sucrose and cholic acid moieties [62]. The functionalization of the PLGA backbone was made through esterification using DCC/NHS reactions, and then sucrose and cholic acid-functionalized PLGA nanoparticles were obtained by nanoprecipitation. Sucrose addition provided colloidal stability to the nanoparticles, demonstrated by the decrease of the negative surface charge. Rieger and collaborators reported a simple method for the preparation of mannose-functionalized PLA NPs [63]. The synthesis approach consisted in the co-nanoprecipitation evaporation of a mannosylated poly(ethylene oxide)-*b*-poly(ϵ-caprolactone) (PEO-*b*-PCL) diblock copolymer with PLA. The amphiphilic copolymers bearing the mannose moieties worked as surface modifiers and were able to specifically bind to MR.

Freichels and co-workers prepared crosslinked hydroxyethyl starch (HES) nanocapsules, which is a hydroxyethylated glucose polymer, functionalized with (oligo)mannose [64]. The preparation of the nanocapsules consisted in interfacial addition of HES with 2,4-toluene diisocyanate (TDI) in inverse miniemulsion. This procedure leaves an amount of non-reacted amine groups that were used to perform the functionalization with three types of mannose molecules: a-d-mannopyranosylphenyl isothiocyanate, 3-O-(a-d-mannopyranosyl)-d-mannose (di-mannose), and α3,α6-mannotriose (tri-mannose). The amine groups on the surface of nanocapsules were used to react directly with mannose isothiocyanate while di- and tri-mannose were coupled through reductive amination. The obtained delivery systems exhibit a specific binding to agglutinin and the presence of a PEG linker showed to increase the interaction to the receptor, due to a higher accessibility of the sugar molecule. 

Kim et al. developed a siRNA delivery system composed of PEI, PEG, and mannose [65]. PEI molecules were used to form the polymer/siRNA polyplex, PEG was used as a stabilizer, and mannose as a targeting ligand for macrophages. Here, two different functionalization methods were performed: one in which PEG and mannose molecules were directly linked to PEI backbone (mannose-PEI-PEG), and another in which mannose chains were conjugated to PEI using a PEG spacer, i.e., mannose was linked to PEG before reaction of mannose-PEG chains to PEI backbone. In these reactions, like the ones described before, α-d-mannopyranosylphenyl isothiocyanate was used for mannosylation and PEG was conjugated to PEI via glutaraldehyde linkage. The researchers also found that the location in which mannose ligands are conjugated affect the cytotoxicity of nanocarriers. Table 1 resumes examples of glycoproteins produced with electrostatic interactions and covalent conjugation strategies identifying the ligand and the target defined for the nanocarriers.

## 5. Macrophages

### 5.1. Functions and Polarization State

The mononuclear phagocytic system, also designated as the reticuloendothelial system, is composed of monocytes in the blood and macrophages in the tissues and is part of the innate immune system. During the hematopoiesis process, mature monocytes circulate for about 8 h, grow, and end up in specific tissues, as macrophages [68]. 

Macrophages are present throughout the body resident in tissues and also motile, known as free or wandering macrophages. They can originate from circulating monocytes, but also from embryonic hematopoietic stem cells or yolk sac [69]. Macrophages play relevant roles in the immune response, as they act in tissue development, inflammation related to pathogens, cancer, and organ transplantation. During phagocytosis, macrophages engulf pathogens, mediated by receptors on macrophage surface that bind to the fragment crystallizable (Fc) region of molecule from the pathogen. This process leads to the formation of a phagosome that merges with the lysosome where the target is digested. Macrophages act as antigen presenting cells, when displaying foreign material or parts of antigens on its surface in association with class II major histocompatibility complex (MHC) molecules. This triggers T-cells, and consequently, the adaptive immunity. Likewise, macrophages can secrete several cytokines involved in the immune response, homeostasis, and inflammation, which modulate their function and surface marker expression [70].

Macrophages are polarized to respond to alterations in their environment, being classified as M1 macrophages and M2 macrophages [71]. Contact with pathogen-associated molecular patterns (PAMPs), such as bacterial lipopolysaccharide (LPS) from *Escherichia coli* (Gram-negative) or peptidoglycan (PGN) from *Staphylococcus aureus* (Gram-positive) drives macrophages polarization towards M1 phenotype, with the ability to elicit proinflammatory response and production of interleukin (IL) 6 (IL-6), IL-12, and tumor necrosis factor-alpha (TNF-α), all pro-inflammatory cytokines. Alternatively, activated macrophages are produced in the presence of the Th2 cytokines IL-4 and/or IL-13, which can lead macrophage polarization to M2, characterized by anti-inflammatory responses and tissue repair abilities [72].

Regulation of macrophage polarization phenotype is reversible and modulates their immune function. An important feature in this mechanism is the expression of the cell surface markers. M1 macrophages overexpress CD80, CD86, and CD16/32, while M2 exhibits more arginase-1 and mannose receptor (CD206).

### 5.2. Macrophage Polarization Mediated by Nanocarriers

To date, several nanocarriers were able to induce inflammatory and immune responses in vitro and in vivo [73,74,75]. Nanocarriers can be internalized by macrophages inducing changes at the cell surface as well as secretion of cytokines and chemokines [76]. Understanding the mechanism of interaction between nanocarriers and macrophages will contribute to an effective design of nanocarriers for a specific therapeutic strategy. Macrophage-mediated therapies are emerging as a promising and effective approach towards the treatment of several diseases. In particular, uptake of nanocarriers by macrophages implies interaction between nanocarriers’ surface and macrophage cell membrane. Therefore, the formed membrane-bound vesicle will have a size, composition, and internal environment according to the internalization, resulting in endosomes, phagosomes, or macropinosomes. In fact, the uptake mechanisms can be described as phagocytosis, micropinocytosis, endocytosis mediated by clathrin or by caveolin, or independent from both [77]. Passive and active targeting approaches can be designed to achieve the intended effect. Size and surface of the nanocarrier govern passive targeting, while for an active targeting the surface of the nanocarrier requires functionalization with a specific ligand towards a particular surface cell receptor. Carbohydrate-coated nanocarriers have been exploited to target mannose receptors expressed in macrophages and dendritic cells (antigen presenting cells, APCs) [51].

Conjugation of ligands at the surface of nanocarriers may modulate the immune system. The use of targeted nanocarriers elicit the maturation of APCs, with alterations at the surface expression of co-stimulatory molecules and in the secretion of cytokines that activate T-cell responses [78,79,80]. Active targeting of nanocarriers towards endocytic receptors present on macrophage surface can be achieved using C-type lectin receptors (CLR) or the mannose receptor CD206.

### 5.3. Mannose Receptor

CD206 or mannose receptor (MR) has the ability to recognize mannosylated or fucosylated glycoproteins and engulf them [81]. This 175 kDa endocytic receptor was first identified in rabbit alveolar macrophages and is a type I transmembrane receptor composed by an extracellular region containing a cysteine-rich (CR) domain that acts as second lectin domain, and a fibronectin type II (FNII) domain that is involved in collagen binding, and multiple C-type lectin-like domains (CTLDs) within a single polypeptide backbone where the binding of sugars terminated in D-mannose, L-fucose, or N-acetyl glucosamine occurs [82]. Based on their structure, CLR are grouped as transmembrane CLRs and soluble CLRs (collectins). Type I transmembrane CLRs include MR and ENDO180 (mannose receptor C type 2), while type II transmembrane CLRs include dendritic cell-specific intracellular adhesin molecule 3 grabbing non-integrin (DC-SIGN), langerin, and macrophage galactose type lectin (MGL) receptors [83].

MR expression is not restricted to resident macrophages and dendritic cells. It was also found on immature monocyte-derived dendritic cells [84], hepatic endothelial cells [85], tracheal smooth muscle cells [86], and kidney mesangial cells, among others [84]. Expression of this receptor is modulated by cytokines, immunoglobulin receptors, and pathogens [87]. MR synthesis is more rapid in the presence of immunoglobulins IgG2a and IgG2b [88]. Cytokines regulate MR expression as IL-4 [89], IL-13 [90], and IL-10 [91] enhance macrophages receptors expression, while interferon-ɣ (IFN-ɣ) [92] down-regulate MR expression and increase macrophage’s activation.

Macrophages cell surface express about 10–30% of MR at steady state and the remaining 70–90% have an intracellular location. Early endosomes contain MR internalized and are able to send these receptors back to the cell surface through the interaction with the clathrin-mediated endocytic machinery [80]. This mechanism is mediated by small intracellular vesicles (below 0.2 μm) and drive the MR to be recycled to the macrophage membrane or delivered into late endosomes, filled with lysosomal hydrolases. Here, under acidic pH and hydrolase-rich environment, the final degradation of the internalized cargo happens. The ability of nanoparticles to modulate the macrophage state through MR was described for several authors (Table 1). For example, chitin and mannose-coated beads improved the production of tumor necrosis factor-alfa (TNF-α), IFN-ɣ, and IL-12 by murine spleen cells in relation to non-coated beads [93].

MR is also expressed in DCs and actively contributes to antigen recognition and processing. Evidences confer MR an important part in the antigen-internalization mechanism in DCs. For example, bovine serum albumin coated with mannose enhanced the uptake and presentation of this antigen to T cells [94,95].

Macrophages are responsible for the internalization and degradation of pathogens, acting as pattern recognition receptors, given the highly conserved C-type lectin receptors, in a calcium dependent manner. Thus, this first line of defense binds to carbohydrate molecules (e.g., mannose, fucose, and *N*-acetyl glucosamine) present on the surface of a wide variety of pathogens, including *Candida albicans* [81], *Leishmania donovani* [96], and *Mycobacterium tuberculosis* [97].

### 5.4. Mannose Receptor-Targeted Nanocarriers Interactions with Macrophages

**Figure 4:**
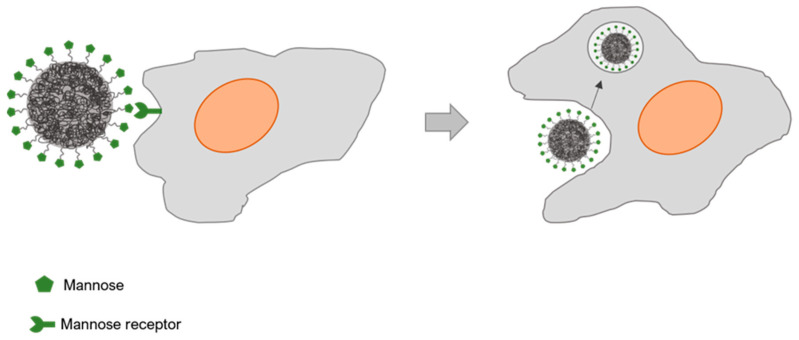
Glyconanoparticle interaction with macrophages through receptor mediated endocytosis mechanism.

Targeting MR in macrophages using polysaccharides or glycoproteins containing mannose or fucose residues has been exploited to develop nanocarrier-based macrophage-mediated therapies [98]. Mannose-based glycopolymers exhibited an increased internalization by macrophages in comparison to galactose-containing glycoprolymers [99]. Given the variability of ligand–target interaction according to the activation and differentiation state of macrophages, studies should consider various types of carbohydrate moieties. The design of the nanocarriers should also pay attention to their surface charge, as it affects macrophage binding affinity. Anionic sialic acid is present on macrophages surface and enhances phagocytosis of positively charged nanocarriers [100]. Nanocarriers coated with albumin, folic acid, or cholesterol are easily internalized by caveolin-mediated endocytosis which prevents lysosomal degradation. However, mechanisms of uptake are interchangeable and blocking a path may “open” another endocytic path, which poses a challenge in the design of a nanocarrier (Figure 4) [5,101].

#### 5.4.1. Mannose Receptor-Targeting Nanocarriers towards Infection Resolution

Macrophages are host cells of many intra-cellular pathogens (bacteria, parasites, and virus) causing infectious diseases that could be managed with nanocarriers targeting MR. Recent examples of carbohydrate-based polymeric nanocarriers towards macrophages are described and shown in Table 2.

Tuberculosis is the bacterial infection responsible for more deaths worldwide. The treatment regimen involves oral administration of rifampicin, isoniazid, pyrazinamide, and ethambutol for long periods, usually over six months. The completion rate is highly dependent of patient compliance, but interruptions may occur due to adverse side-effects. Hence, new therapeutic approaches which are more efficient, with less side-effects and shorter duration of treatment are envisaged [102]. Aminoglycoside antibiotics are used against mycobacterial infections, but usually are not highly membrane permeable eliciting adverse side effects. Chitosan nanoparticles loaded with aminoglycoside were produced with dextran sulphate as counter ion to shield the positive charge of the antibiotic. In vivo results showed effective killing of intracellular *M. tuberculosis* upon oral administration of antibiotic-loaded nanocarriers [103]. Isoniazid, an anti-tuberculostatic agent, was incorporated in mannosylated gelatin nanoparticles. Macrophages were effectively targeted by these nanoparticles, as assessed by flow cytometry [104]. For rifampicin, several examples of nanocarriers have been described. Rifampicin was loaded in dendrimers able to enhance alveolar macrophage uptake and drug release at pH 5 [105], and also in flower-like polymeric micelles which surface was modified with hydrolyzed galactomannan [106]. The latter combined mannose and galactose were both recognized by CLRs. A complex nanocarrier based on poly(epsilon-caprolactone)-*b*-poly(ethylene-glycol)-*b*-poly(epsilon-caprolactone) flower-like polymeric micelles (PMs) coated with chitosan or GalM-h/chitosan was produced allowing higher intracellular levels of rifampicin in murine macrophages, relative to its free and chitosan-loaded forms.

The protozoa Leishmania is the causative agent of several infectious diseases upon invading macrophages in the liver and spleen (visceral leishmaniasis) or in the skin (cutaneous leishmaniasis). Leishmaniasis remains endemic in developing countries and without proper treatment leads to death. Pentavalent antimonials were the first anti-leishmanial agents used, but given their toxicity, treatment evolved to amphotericin B, miltefosine, pentamidine, primaquine, paromomycin, and even natural compounds (e.g., amarogentin and andrographolide) [107]. Treatment is hampered by the intracellular localization of the protozoa inside the phagolysosome. The US FDA-approved poly(d,l-lactide-coglycolide) (PLGA) polymer was functionalized with carbohydrate moieties (mannose, mannan, and mannosamine) to identity the most effective in targeting macrophages infected with *Leishmania*. In vitro data obtained with murine primary macrophages evidenced the immune-modulatory properties of the nanocarriers, with activation of macrophages and production of pro-inflammatory cytokines, upon clathrin-mediated endocytosis. Amphotericin B-loaded on mannan-functionalized PLGA nanocarriers confirmed in vivo efficacy in relation to Fungizone^©^ alone, in a visceral leishmaniasis model [66]. MR was targeted by coating polyanhydride nanoparticles with carbohydrates (galactose and di-mannose) by Chavez-Santoscoy and co-workers [108]. The designed nanocarriers increased surface expression of markers in alveolar macrophages, enhanced the expression of MR, and promoted production of pro-inflammatory cytokines (IL-1b, IL-6, and TNF-a). Curcumin-loaded mannosylated chitosan nanoparticles improved the drug mean residence time within infected macrophages [109]. Effective endocytosis mediated by MR lead to better pharmacokinetic parameters. 

Targeted mannose-coated gelatin nanoparticles were produced to enhance therapeutic efficacy of didanosine towards human immunodeficiency virus [110]. Higher uptake by alveolar macrophages was observed with the mannose coating, and in vivo biodistribution studies revealed the presence of the nanocarriers in the spleen, lymph nodes, and lungs. Lamiduvine delivery towards HIV was improved with the incorporation in stearate-g-chitosan oligosaccharide polymeric micelles. The nanocarrier led to high internalization and low cytotoxicity in viral transfected cells [111]. Another antiretroviral drug, zidovudine, was incorporated within sialic acid and mannose dual-coated poly(propyleneimine) dendrimer [112]. This nanocarrier produced less cell toxicity and hemolysis, most probably related to the zidovudine-sustained release and enhanced internalization by macrophages. In vivo biodistribution revealed targeting to sialo-adhesin and carbohydrate receptors in the lymph nodes. 

#### 5.4.2. Mannose Receptor-Targeting Nanocarriers towards Tumor-Associated Macrophages

Macrophages accumulate in the tumor microenvironment, being designated as tumor-associated macrophages (TAM). These represent the major contribution of tumor immune escape, angiogenesis, growth, and metastasis [113]. Mannosylated nanocarriers can modulate macrophage polarization from M2 phenotype to the M1 phenotype enhancing anti-tumor immunity. Delivery of Toll-like receptor (TLR) agonists reset TAM polarization towards an antitumor M1 phenotype. Rodell and co-workers produced b-cyclodextrin nanoparticles containing a TLR7/8 agonist that reprogrammed TAM and, as a consequence, efficiently controlled tumor growth [114].

MR targeting can also contribute to improve gene delivery efficiency, by improving transfection and tissue specificity. Reeducation of TAM can be accomplished with delivery of siRNA, miRNA, or mRNA using mannosylated nanoparticles [115,116]. Likewise, chitosan nanoparticles allowed to deliver therapeutic DNA by MR-mediated endocytosis [117]. Experimental data highlights less cytotoxicity, improved gene transfection, and induction of IFN-γ production upon IL-12 gene delivery, in comparison to plain chitosan nanocarriers. IL-12-based gene delivery can be applied for cancer immunotherapy, as it elicits a Th1-type immunity and also cell-mediated immunity.

Instead of only modulating TAM polarization to control cancer progression, it is also possible to completely neutralize or kill them, with the delivery of cytotoxic compounds using TAM-targeted nanoparticles [118].

Nanoparticle-based immunotherapies represent a promising approach to target tumor environment, in particular TAM, instead of aiming for the tumor cells, preventing immune-mediated adverse-effects. Another application could be cancer vaccination by targeting immune cells in the lymph node.

#### 5.4.3. Mannose Receptor-Targeting Nanocarriers towards Prevention Approaches

Oral delivery is the preferred route for drug/bioactive compounds administration, due to effects both at a local and systemic level, minimal invasiveness, and cost-effectiveness [119,120]. However, a question of bioavailability and efficacy emerges when these immunomodulatory compounds are orally administered in its free form. This can be attributed to compound degradation due to pH variation and enzymatic activity in the gastrointestinal (GI) tract or poor permeability across intestinal biological membranes [119,121,122]. Delivery systems such as carbohydrate-functionalized polymeric nanoparticles are able to provide protection from degradation in the GI tract, increase absorption by the intestinal epithelium due to its mucoadhesive properties (e.g., PLGA, chitosan, and alginate) and cell or tissue-targeted delivery and sustained release [121,123,124,125,126]. Gentamicin (GM) is an antibiotic that can only be administered in parenteral form or in topical formulations, and it cannot be orally administered due to enzymatic degradation and poor bioavailability. However, when GM was encapsulated in chitosan-functionalized PLGA nanoparticles and orally given to healthy rabbits, it not only reached the GI tract, as it was able to cross the membrane entering the blood stream [127]. Based upon these findings the authors concluded that biodegradable chitosan-functionalized PLGA nanoparticles are potential candidates for GM oral delivery. Furthermore, these polysaccharide polymers-based nanoparticles show unique physicochemical properties, namely, biocompatibility, biodegradability, non-toxicity, and low cost [128,129].

Immunomodulators targeting myeloid cells, particularly macrophages, are a proven strategy to improve the host immunological status and immune response. Several studies show that oral immunostimulation with bioactive compounds can be an effective prophylactic strategy to prevent infectious disease or curtail its effects [130,131,132]. As already mentioned, macrophages perform critical roles in innate immune response, including inflammation and tissue repair, pathogen elimination, and coordination of the adaptive immune response. Cell surface receptors that recognize polysaccharide residues such as mannose, galactose, or *N*-acetylglucosamine residues are paramount for macrophage activation and response. Carriers comprising a matrix of polysaccharide moieties, or surface ligands composed of carbohydrates, are suitable candidates for macrophage targeting or stimulation. A chitosan nanoparticle functionalized with a high molecular weight ulvan polysaccharide, activated Senegalese sole (*Solea senegalensis*) macrophages and triggered a stronger immune response than the ulvan extract free form. Ulvan is a complex polysaccharide composed of glucuronic acid and sulphated rhamnose, known to activate and induce a potent stimulating effect on macrophage oxidative burst [133]. It was hypothesized that ulvan stimulating properties improved in the chitosan/ulvan nanoparticles possibly due to particle endocytic uptake by macrophages [134]. Particle size is an important feature for cell uptake: when comparing microparticles to nanoparticles, the latter is generally having higher cell internalization rates, and thus can be utilized to target cellular and intracellular receptors due to their smaller size and mobility [135]. Furthermore, several studies explored mannose-functionalized nanoparticles recognition by the macrophage MR as a way to stimulate macrophages [67,136]. 

The potential to use orally delivered carbohydrate-functionalized polymeric nanoparticles to target macrophages is recognized, mostly because of the unique structural features of polysaccharides referred above. As research progresses in the field of nutraceuticals, these glyconanoparticles seem to be a highly suitable delivery system for biologically active compounds targeting macrophages.

## 6. Future Perspectives 

Further application of carbohydrate-functionalized polymeric nanoparticles depends on more efficient production methods and improved selectivity towards macrophages or other defined targets (Table 3). The design should consider drug release rate to assure rapid release of the cargo at the target site. The amount of loaded cargo is also crucial, since a balance needs to be achieved between high capacity and safety of the total administered dose. Altogether, the product should be scalable and cost-effective to attract investors and industries. However, not all these requirements are currently met. In fact, the production methods are hardly reproducible, as the molecular weight, functional groups, and purity of polymers depends on the source and batch. More knowledge on the mechanism of interaction between glyconanoparticles and targeted macrophages will certainly allow to optimize these parameters and obtain a product for further translation. In fact, the potential of the carbohydrate-functionalized nanoparticles is highlighted by the increasing number of patents found on the World Intellectual Property Organization and recently discussed by Patil and Deshpande [73].

## 7. Conclusions

Carbohydrates play a fundamental role in many aspects of receptor-mediated delivery and therapies. The insertion of carbohydrates in biodegradable polymeric nanoparticles enhances their biocompatibility and favors their use for biomedical applications. In this review, we focused on the preparation methods and use of carbohydrate-functionalized polymeric nanoparticles for macrophage targeting. The sugar moieties present in these nanocarriers are able of specifically interacting with receptors at the surface of macrophage cells and trigger immune responses. The study of this interaction makes the development of new macrophage-mediated therapies possible, with the mannose receptor binding being the most exploited, due to its abundant expression in dendritic cells and increased internalization. Mannose-targeting nanocarriers have shown to be effective in increasing the production of pro-inflammatory cytokines, in infection resolution, modulate tumor-associated macrophages’ polarization, and improving nutraceuticals oral administration.

## Figures and Tables

**Table 1:** List of developed carbohydrate-functionalized polymeric nanoparticles.

Polymeric Nanocarrier Composition	Carbohydrate Ligand	Functionalization Strategy	Target Tissue/Cells	Ref
Cholic acid and PEG	Galactose	*N,N*′-dicyclohexyl carbodiimide reaction	Liver-specific delivery	[65]
PLGA NPs	Galactose Glucose Mannose	*N,N*′-diisopropylcarbodiimide/NHS reaction	-	[61]
PLGA NPs	Sucrose Cholic acid	DCC/NHS reactions	-	[62]
PLA and PEO-*b*-PCL diblock copolymer NPs	Mannose	Nanoprecipitation-evaporation approach	Mannose receptors	[63]
Hydroxyethyl starch nanocapsules	MannoseDimannose Trimannose	Mannose: -Amine to isothiocyanate group reaction Dimannose and trimannose: -Reductive amination	Agglutinin (mannose receptor)	[64]
PEI-PEG NPs	Mannose	Binding of mannose to PEI-PEG NPsBinding of mannose to PEI NPs via a PEG spacer	Macrophage cells	[65]
PLGA NPs	Mannose Mannan Mannoseamine	DCC/NHS/EDA reaction	Macrophages *Leishmania*-infected mice	[66]
6-Amino-6-deoxy-curdlan	Mannose	Amine to isothiocyanate group reaction	Mouse peritoneal macrophages	[67]

**Table 2:** Mannose receptor-targeting nanocarriers towards infection resolution.

Composition	Carbohydrate	Cargo	Advantages	Ref
Chitosan, dextran sulphate	-	Aminoglycoside	Oral administration allowed effective killing of intracellular *M. tuberculosis*	[103]
Gelatin	Mannose	Isoniazid	Effective targeting of macrophages	[104]
Poly(epsilon-caprolactone)-*b*-poly(ethylene-glycol)-*b*-poly(epsilon-caprolactone) and chitosan	Galactomannan	Rifampicin	Improved cellular internalization in murine macrophages	[106]
PLGA	Mannose, mannan and mannosamine	Amphotericin B	Improved in vivo efficacy against visceral leishmaniasis	[66]
Polyanhydride	Galactose and di-mannose	-	Increase production of pro-inflammatory cytokines	[108]
Chitosan	Mannose	Curcumin	Enhanced the drug residence time within infected macrophages	[109]
Gelatin	Mannose	Didanosine	Improved uptake by alveolar macrophages, and in vivo distribution mainly in the lungs, spleen and lymph nodes	[110]
Stearate-g-chitosan	Oligosaccharide	Lamiduvine	High cellular uptake with low toxicity in viral infected cells	[111]
Sialic acid and poly(propyleneimine)	Mannose	Zidovudine	Low cell toxicity and in vivo biodistribution on the lymph nodes	[112]

**Table 3:** A resume of the advantages and limitations of mannose receptor-targeting polymeric nanocarriers.

Advantages	Limitations
Surface chemistry can be controlled to reduce impact in the nanoparticles toxicity, immunogenicity, and biodistribution	Production of heterogeneous populations
Improved pharmacokinetics/pharmacodynamics profile	Nanoparticles stability during storage, in contact with blood and tissues
Effective internalization in targeted cells	Scale up and time of production, particularly for functionalized nanoparticles
Site-specific delivery with reduced side-effects	
High binding affinity for targeted cells	
